# Retroperitoneal anastomotic hemangioma with significant steatosis: A case report and literature review

**DOI:** 10.1097/MD.0000000000043712

**Published:** 2025-08-08

**Authors:** Luxin Tan, Wei Wang, Tao Wu, Suxia Wang

**Affiliations:** a Laboratory of Electron Microscopy, Pathological Center, Peking University First Hospital, Peking University, Beijing, P.R. China; b Department of Pathology, Pathological Center, Peking University First Hospital, Peking University, Beijing, P.R. China; c Department of Gastrointestinal Surgery, Peking University First Hospital, Peking University, Beijing, P.R. China.

**Keywords:** anastomosing hemangioma, case report, hemangioma, retroperitoneal, steatosis

## Abstract

**Rationale:**

Anastomosing hemangioma (AH) is a rare variant of hemangioma that was proposed in 2020. It is primarily located in the genitourinary tract, though it can occasionally be found in the retroperitoneum. Typically, classic hemangiomas exhibit a red cavernous appearance on the cut surface. This case presents a retroperitoneal AH characterized by marked fatty changes, displaying gross features that differ significantly from those of conventional hemangiomas. This rare variant of hemangioma is rarely reported.

**Patient concerns:**

A 59-year-old Chinese female patient was discovered to have an anechoic nodule located beneath the lower pole of the right kidney during ultrasonography performed as part of a physical examination.

**Diagnoses and interventions:**

Laparoscopic surgery was conducted following surgical evaluation, and the histopathological diagnosis confirmed hemangioma.

**Outcomes:**

The patient has been monitored for 30 months, during which no recurrence or metastasis of the tumor has been observed.

**Lessons:**

Retroperitoneal AHs are often located in the perirenal fat space. Preoperative imaging often suggests a diagnosis of paraganglioma due to the location and imaging findings, indicating that accurate identification of the mass type by imaging diagnosis is still challenging. AH with significant steatosis cannot be accurately diagnosed by gross examination and histology alone, while immunohistochemistry and molecular pathology can assist in the diagnosis.

## 1. Introduction

Initially, the nomenclature of vascular anomalies was clinically descriptive and nonuniform, which led to diagnostic confusion until Mulliken and Glowacki proposed a standardized nomenclature for vascular lesions in 1982 and classified vascular malformations into 2 groups based on pathological attributes: vascular tumors (hemangiomas) and vascular malformations.^[1]^ Compared with vascular malformations, the main difference in pathological features of hemangioma is the abnormal proliferation of vascular endothelial cells. Hemangiomas typically present as red masses with limited growth. Based on the clinical manifestations and the caliber of the affected blood vessels, they are classified into capillary hemangiomas, cavernous hemangiomas, venous hemangiomas, and other variants.^[2]^ Anastomosing hemangioma (AH), a new variant proposed in the 2020 WHO classification of soft tissue tumors, primarily occurs in the genitourinary tract but can also be found in various organs and soft tissues, including the liver, gastrointestinal tract, breast, mesentery, and peritoneum.^[3,4]^ In this paper, we report a case of retroperitoneal AH, whose imaging features, gross tumor characteristics, and pathological findings were inconsistent with those of traditional hemangiomas.

## 2. Case presentation

**Figure 1.**
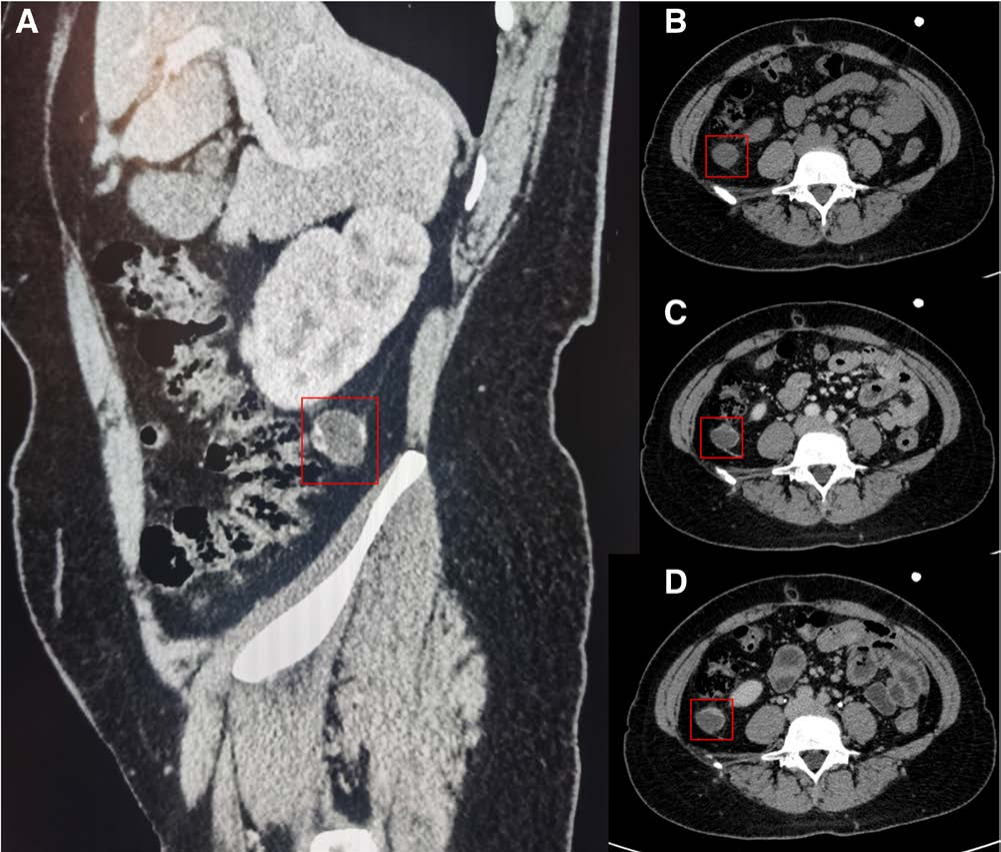
The abdominal CT scan (A) revealed a low-density cystic lesion measuring 2.8 cm × 2.4 cm in the perirenal space, located posterior to the lower pole of the right kidney. Multi-phase enhancement of the abdomen and pelvis (B–D) demonstrated significant enhancement of the cyst wall, which exhibited uneven thickness, along with the presence of cords surrounding the cyst wall.

A 59-year-old Chinese female patient was discovered to have an anechoic nodule located beneath the lower pole of the right kidney during ultrasonography performed as part of a physical examination. The patient reported no symptoms, including abdominal pain, abdominal distention, altered bowel habits, or hematochezia. Moreover, she had no significant medical, cancer, or family history. Laboratory tests, including routine blood examinations and serum electrolytes, returned normal results. Among the tumor markers, carbohydrate antigen 724 (CA724) was slightly elevated at 8.60 U/mL, while carcinoembryonic antigen (CEA), carbohydrate antigen 199 (CA199), and carbohydrate antigen 242 (CA242) remained within normal ranges. An abdominal CT scan revealed a round low-density lesion adjacent to the ascending colon, the nature of which was indeterminate. Multi-phase enhancement of the abdomen and pelvis indicated a cystic mass in the right perirenal space, also of indeterminate nature, with schwannoma being considered as a differential diagnosis (Fig. 1). Ultimately, laparoscopic surgery was conducted following surgical evaluation, and the histopathological diagnosis confirmed hemangioma. Informed consent was obtained from the patients for the evaluation and publication of their data in this study, and follow-up was conducted by telephone. The patient has been under surveillance for 30 months and is currently well, during which time no tumor recurrence or metastasis has been observed. She told us that surgical removal of the mass had been effective in reducing her anxiety.

## 3. Findings of the operation and pathology

**Figure 2.**
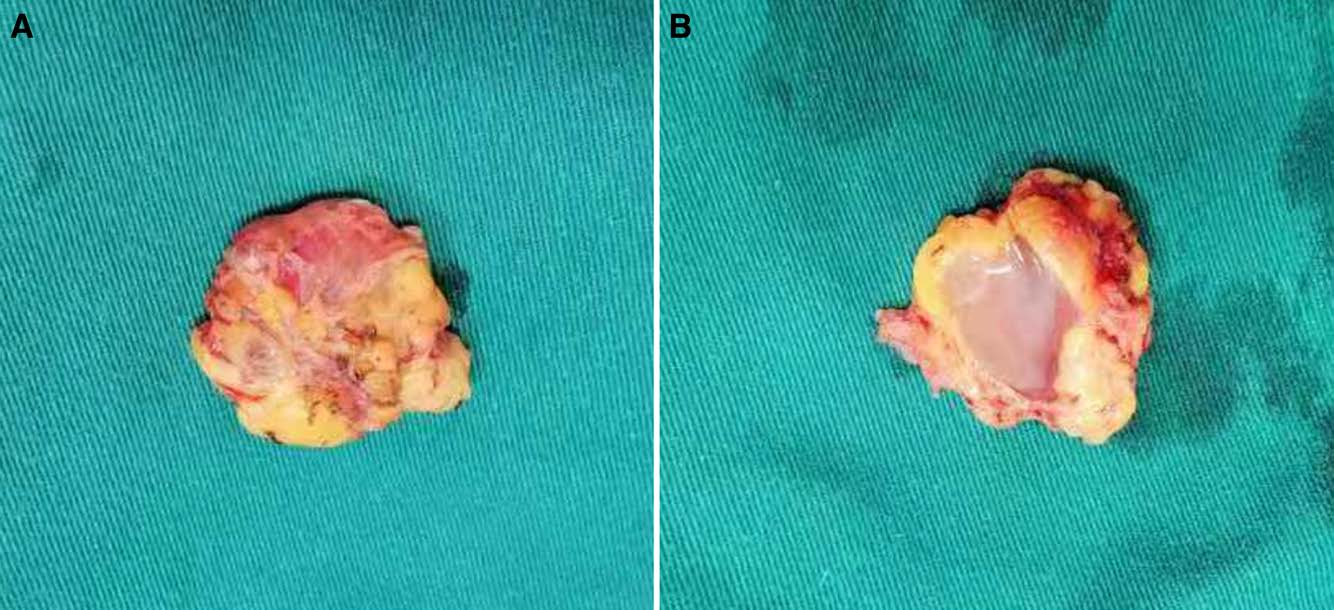
The tumor had an intact surface capsule (A) and a glia-like cut surface (B).

**Figure 3.**
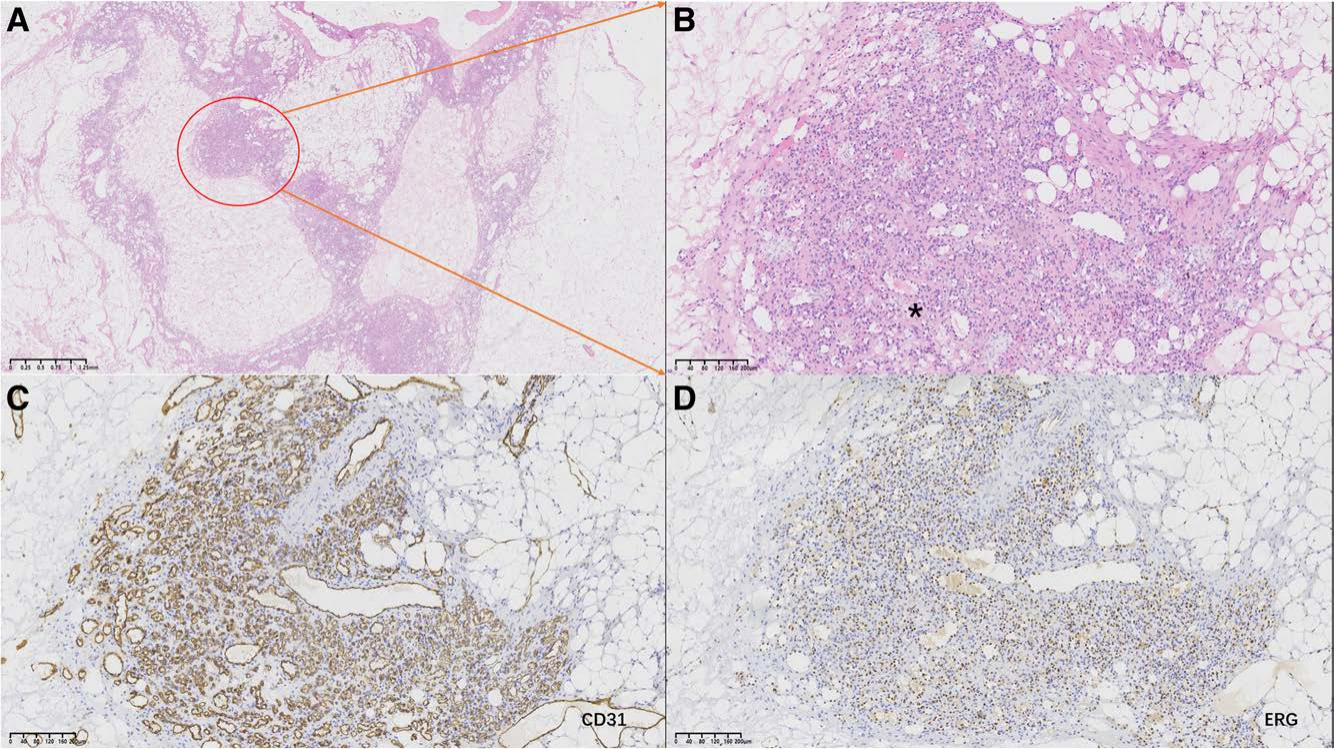
(A) At low magnification, H&E staining showed 2 tissue components, including proliferating tumor cells of small hemangiomas and adipocytes. (B) At high magnification, the endothelial cells appeared flat to cuboidal, exhibiting a boot-staple appearance on the surface of the protruding lumen. Numerous red blood cells and fibrin deposits were observed within the vessel (*). Immunohistochemistry showed the tumor cells expressed endothelial cell markers, which showed positive CD31 (C) and ERG (D).

During the operation, an oval mass measuring 3 cm in diameter was located 5 mm from the lower pole of the right kidney. The surface capsule remained intact, and the cut surface exhibited a gray-white, glia-like appearance (Fig. 2). At low magnification, tumor cells displayed small, angioma-like proliferations, surrounded by areas of fatty degeneration, with some adipocytes demonstrating degenerative changes. At high magnification, there was a dense proliferation of small blood vessels with variable lumen sizes and focal intercellular fibrosis. The endothelial cells appeared flat to cuboidal, exhibiting a boot-staple appearance on the surface of the protruding lumen. The cell morphology was bland, and mitotic figures were infrequent. Immunohistochemical analysis revealed that the tumor cells were positive for CD31, ERG, and Syn, while negative for CKpan. Additionally, the peripheral adipocytes tested positive for S-100 (Fig. 3).

## 4. Discussion

AH is a rare subtype of capillary hemangioma first defined by Montgomery et al in 2009.^[5]^ 2017 Bean conducted a DNA sequencing analysis on 13 patients with confirmed AH. In 9 of the 13 patients (69%), a somatic mutation occurred at codon 209 of the GNAQ gene, a common mutation hotspot in uveal melanoma, blue nevi, and various congenital vascular proliferations. Therefore, it was hypothesized that AH is a clonal neoplastic lesion.^[6]^

The PUBMED database was searched with anastomosing hemangioma as the keyword. Case reports with complete clinical information, including imaging data before surgical resection and histopathological pictures after surgical resection, were selected. A summary of case reports regarding the surgical resection of angiomatosis hemangiomas (AH) over the past 5 years is presented in Table 1. Currently, the majority of reported cases of retroperitoneal reticular hemangiomas following surgical resection are located in Asia, predominantly affecting middle-aged and elderly women.^[8–11,13]^ Due to the deep-seated nature of these tumors within the retroperitoneal perirenal space, most patients exhibit no clinical symptoms or signs, with the tumors often discovered incidentally during routine physical examinations, with only a few patients accompanied by abdominal pain.^[7]^ The maximum diameter of these tumors varies significantly, typically ranging from 2 to 4 cm. While most tumors are single nodules, instances of multiple nodules have also been documented.^[12]^ Notably, preoperative imaging often suggests a diagnosis of paraganglioma or a benign neurogenic tumor, particularly in the absence of a specific medical history. Conversely, when a patient has a prior history of cancer, the possibility of metastasis must be prioritized.^[11,12]^ This underscores that while preoperative imaging can assist in distinguishing between benign and malignant retroperitoneal masses, it does not provide definitive identification of the specific mass type. The clinical and preoperative details of the case presented here align with those of previously reported cases; the patient, a middle-aged woman, was found to have a retroperitoneal space-occupying lesion during a routine physical examination, with preoperative imaging suggesting a diagnosis of schwannoma.

**Table 1:** Case reports of isolated retroperitoneal AH surgically resected in the last 5 years.

Authors	Countries	Gender/age	Location	Maximum diameter (cm)	Symptom	Imaging diagnosis	Follow-up (m)
Muñoz-Caicedo et al^[7]^	Colombia	M/55y	Adjacent to the left renal vein	4.5	Chronic abdominal pain	Exophytic renal angiomyolipoma, pheochromocytoma (hypertensive patient), or AH	NA
Ishido et al^[8]^	Japan	F/67y	Below the left renal vein	3.5	No	Benign neurogenic tumor or retroperitoneal cavernous hemangioma	15
Zheng et al^[9]^	China	F/74y	The left renal vein	2.6	No	Benign neurogenic tumor	2
Kishida et al^[10]^	Japan	F/75y	The upper pole of the left kidney	2.6	No	Paraganglioma, solitary fibrous tumor, or schwannoma	NA
Jayaram et al ^[11]^	India	F/53y	Adjacent to the left common iliac vessel	4	No	Paraganglioma or a metastatic lesion (history of breast cancer)	NA
Burton et al^[12]^	Canada	M/68y	In the left perirenal fat, right retrocaval, right iliac and bilateral adrenal nodules (multifocal)	1.5–3.7	NA	metastatic lesions (history of papillary renal carcinoma)	NA
Xue et al^[13]^	China	F/64	In the left retroperitoneal space	10.8	No	Paraganglioma	24

AH = anastomosing hemangioma.

A summary of retroperitoneal angiolipomas (AH) with more detailed pathological features is presented in Table 2. Retroperitoneal AHs typically manifest as solid or cystic masses, exhibiting a hemorrhagic, mahogany-colored, spongy appearance that may or may not be encapsulated. The tumor comprises anastomosing capillary-sized vessels interspersed with scattered hobnail endothelial cells. These cells are generally bland, displaying minimal cellular atypia, and mitotic figures are infrequent. Immunohistochemical analysis revealed that the tumor cells expressed endothelial cell markers, including CD31, CD34, and ERG. Notably, in this case, the cut surface of the tumor differed from that of common hemangiomas, appearing grayish-white with a glial-like texture. This atypical presentation may be associated with significant fatty changes within the tumor.

**Table 2:** Pathological feature of retroperitoneal AH in 5 cases.

Features	Ishido et al^[8]^	Zheng et al^[9]^	Kishida et al^[10]^	Jayaram et al^[11]^	Xue et al^[13]^
Gross appearance	A solid mass with mahogany-colored, spongy appearance cut surface	A mahogany brown mass with ill-defined borders	A brownish-colored solid mass	An oval mass with a cystic hemorrhage area, a dark brown-colored cut surface	A well-defined and irregular, cystic and solid mass
Tumor capsule	Yes	No	NA	Yes	Yes
Cellular atypia	No	No	No	Minimal	No
Mitosis	No	No	No	No	Scarce
Degeneration	No	No	Fatty change	Fatty change	No

AH = anastomosing hemangioma.

Among the reported cases, AH is most commonly associated with fatty changes.^[10,11]^ However, there was no significant difference in preoperative imaging between AH with fatty changes and other forms of AH, and paraganglioma or schwannoma were the primary considerations in the imaging diagnosis. In pathological diagnosis, AH with fatty changes is primarily differentiated from lipoma and well-differentiated liposarcoma. Lipoma consists of mature adipose tissue that lacks atypia, rendering it indistinguishable from normal adipose tissue based on cell morphology or immunohistochemistry. Lipomas can be further classified into various types according to morphological differences, including myelolipoma, intermuscular lipoma, pleomorphic lipoma, and several other variants.^[14]^ Angiolipoma, one of the variants, can exhibit vascular components alongside adipose tissue proliferation. Notably, angiolipomas are more prevalent after puberty and typically present on the trunk or limbs. They often occur in multiple forms and are associated with pain, allowing for differentiation from AH based on these characteristic clinical manifestations.^[15]^ Liposarcoma is the most prevalent soft tissue sarcoma in adults, frequently occurring in the retroperitoneum. The most common subtype of liposarcoma is well-differentiated liposarcoma, which exhibits gross and microscopic characteristics akin to those of ordinary lipoma. Lipoblasts serve as the key diagnostic morphological feature of liposarcoma. Additionally, the amplification of MDM2 or CDK4, detected through FISH, can aid in the diagnosis of liposarcoma.^[16]^

In this case, the AH was associated with marked steatosis. On examination of the specimen, the gray-white cut surface and glia-like appearance of the mass were also inconsistent with the conventional angioma-like morphology. Although the immunohistochemical findings supported the diagnosis of AH, unfortunately, we did not have molecular pathological findings to support it. AH is a rare subtype of capillary hemangioma, and it is even rarer to occur in the retroperitoneum. More cases need to be collected for further study of retroperitoneal AH, but the collection of rare cases requires a lot of time and multi-center support.

## 5. Conclusion

AH is a variant of hemangioma that was proposed in 2020. A close arrangement of capillaries characterizes it and occasionally occurs in the retroperitoneum. Among retroperitoneal AH cases, those located in the perirenal fat space are the most common. Classic hemangiomas typically exhibit a red cavernous appearance on the cut surface. AH, which may present with significant fatty changes, is generally atypical and is primarily differentiated from lipoma and liposarcoma through histopathological examination. Immunohistochemical staining and molecular pathology are helpful for the diagnosis. Immunohistochemical staining for CD31 and ERG and molecular pathology revealed a somatic mutation at codon 209 of the GNAQ gene usually supports the diagnosis of hemangioma. Due to the location and imaging findings, preoperative imaging often suggests a diagnosis of paraganglioma, highlighting the need for careful imaging analysis to distinguish between benign and malignant retroperitoneal masses. However, the precise identification of the mass type, including AH, remains challenging.

## Acknowledgments

We would like to express our gratitude to the patient for granting permission to use their clinical data in this paper and for the publication of this research.

## Author contributions

**Conceptualization:** Tao Wu, Suxia Wang.

**Data curation:** Wei Wang.

**Resources:** Wei Wang.

**Writing – original draft:** Luxin Tan.

**Writing – review & editing:** Tao Wu, Suxia Wang.
